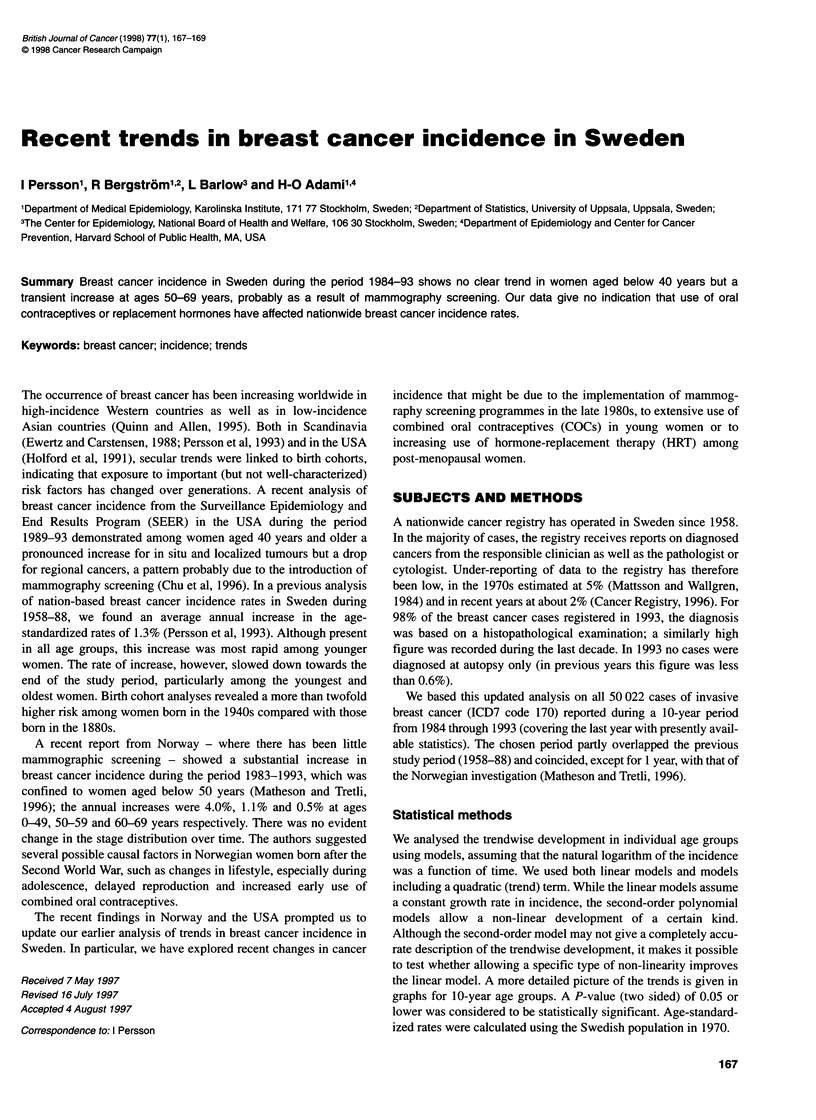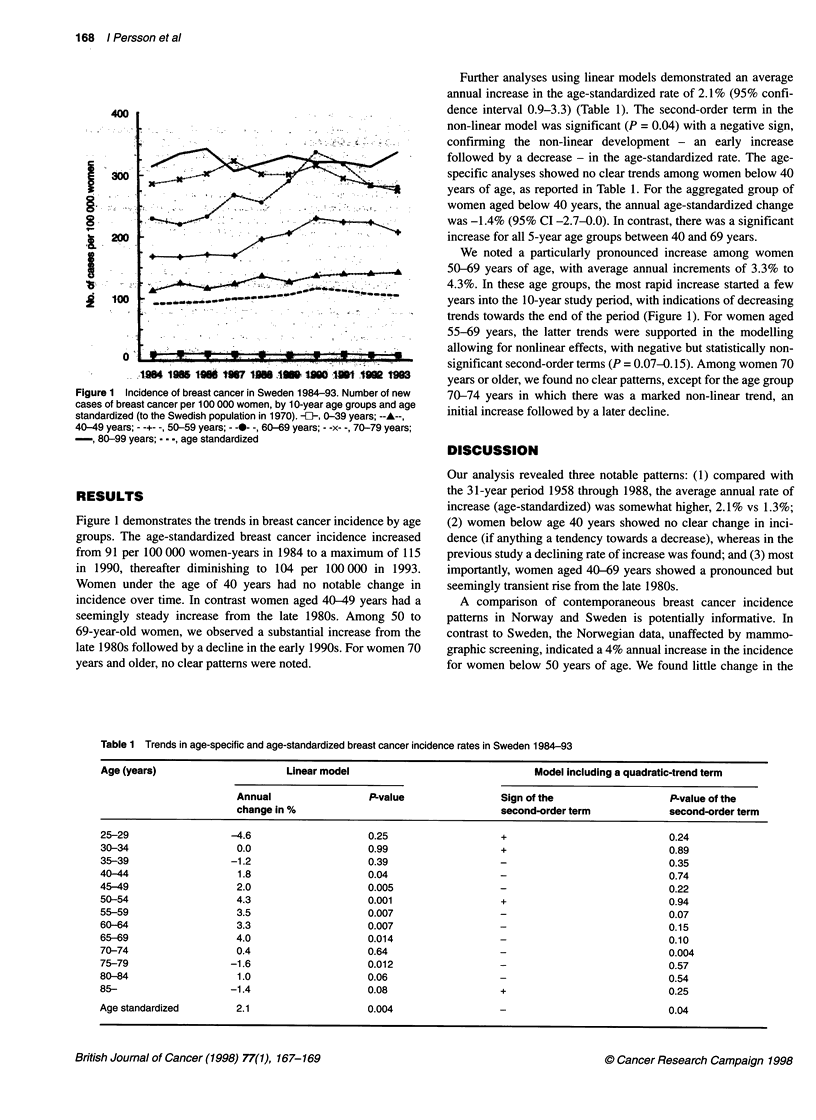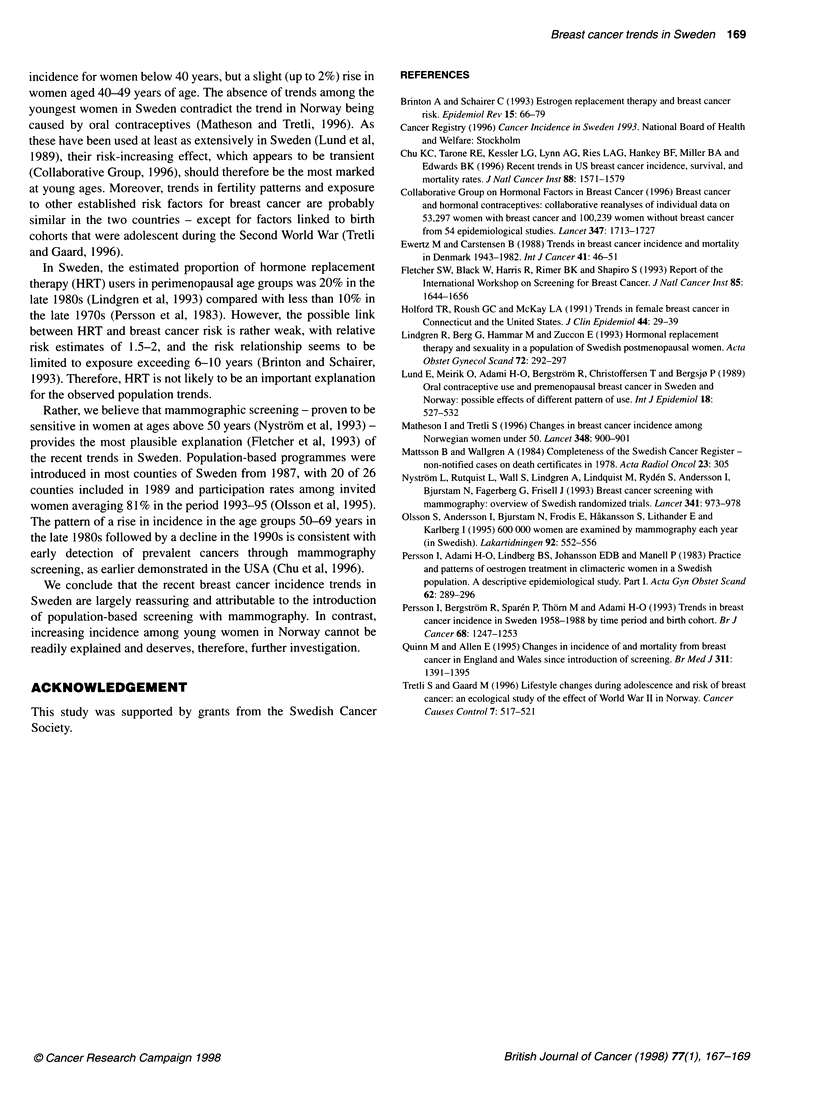# Recent trends in breast cancer incidence in Sweden.

**DOI:** 10.1038/bjc.1998.26

**Published:** 1998

**Authors:** I. Persson, R. BergstrÃ¶m, L. Barlow, H. O. Adami

**Affiliations:** Department of Medical Epidemiology, Karolinska Institute, Stockholm, Sweden.

## Abstract

Breast cancer incidence in Sweden during the period 1984-93 shows no clear trend in women aged below 40 years but a transient increase at ages 50-69 years, probably as a result of mammography screening. Our data give no indication that use of oral contraceptives or replacement hormones have affected nationwide breast cancer incidence rates.


British Journal of Cancer (1998) 77(1), 167–169
© 1998 Cancer Research Campaign

### Recent trends in breast cancer incidence in Sweden

**I Persson[1], R Bergström[1,2], L Barlow[3] and H-O Adami[1,4]**

[1]Department of Medical Epidemiology, Karolinska Institute, 171 77 Stockholm, Sweden; [2]Department of Statistics, University of Uppsala, Uppsala, Sweden; [3]The Center for Epidemiology, National Board of Health and Welfare, 106 30 Stockholm, Sweden; [4]Department of Epidemiology and Center for Cancer Prevention, Harvard School of Public Health, MA, USA

**Summary** Breast cancer incidence in Sweden during the period 1984–93 shows no clear trend in women aged below 40 years but a transient increase at ages 50–69 years, probably as a result of mammography screening. Our data give no indication that use of oral contraceptives or replacement hormones have affected nationwide breast cancer incidence rates.

**Keywords:** breast cancer; incidence; trends

The occurrence of breast cancer has been increasing worldwide in high-incidence Western countries as well as in low-incidence Asian countries (Quinn and Allen, 1995). Both in Scandinavia (Ewertz and Carstensen, 1988; Persson et al, 1993) and in the USA (Holford et al, 1991), secular trends were linked to birth cohorts, indicating that exposure to important (but not well-characterized) risk factors has changed over generations. A recent analysis of breast cancer incidence from the Surveillance Epidemiology and End Results Program (SEER) in the USA during the period 1989–93 demonstrated among women aged 40 years and older a pronounced increase for in situ and localized tumours but a drop for regional cancers, a pattern probably due to the introduction of mammography screening (Chu et al, 1996). In a previous analysis of nation-based breast cancer incidence rates in Sweden during 1958–88, we found an average annual increase in the age-standardized rates of 1.3% (Persson et al, 1993). Although present in all age groups, this increase was most rapid among younger women. The rate of increase, however, slowed down towards the end of the study period, particularly among the youngest and oldest women. Birth cohort analyses revealed a more than twofold higher risk among women born in the 1940s compared with those born in the 1880s.

A recent report from Norway – where there has been little mammographic screening – showed a substantial increase in breast cancer incidence during the period 1983–1993, which was confined to women aged below 50 years (Matheson and Tretli, 1996); the annual increases were 4.0%, 1.1% and 0.5% at ages 0–49, 50–59 and 60–69 years respectively. There was no evident change in the stage distribution over time. The authors suggested several possible causal factors in Norwegian women born after the Second World War, such as changes in lifestyle, especially during adolescence, delayed reproduction and increased early use of combined oral contraceptives.

The recent findings in Norway and the USA prompted us to update our earlier analysis of trends in breast cancer incidence in Sweden. In particular, we have explored recent changes in cancer incidence that might be due to the implementation of mammography screening programmes in the late 1980s, to extensive use of combined oral contraceptives (COCs) in young women or to increasing use of hormone-replacement therapy (HRT) among post-menopausal women.

*Received 7 May 1997*
*Revised 16 July 1997*
*Accepted 4 August 1997*

*Correspondence to:* I Persson

### SUBJECTS AND METHODS

A nationwide cancer registry has operated in Sweden since 1958. In the majority of cases, the registry receives reports on diagnosed cancers from the responsible clinician as well as the pathologist or cytologist. Under-reporting of data to the registry has therefore been low, in the 1970s estimated at 5% (Mattsson and Wallgren, 1984) and in recent years at about 2% (Cancer Registry, 1996). For 98% of the breast cancer cases registered in 1993, the diagnosis was based on a histopathological examination; a similarly high figure was recorded during the last decade. In 1993 no cases were diagnosed at autopsy only (in previous years this figure was less than 0.6%).

We based this updated analysis on all 50 022 cases of invasive breast cancer (ICD7 code 170) reported during a 10-year period from 1984 through 1993 (covering the last year with presently available statistics). The chosen period partly overlapped the previous study period (1958–88) and coincided, except for 1 year, with that of the Norwegian investigation (Matheson and Tretli, 1996).

### Statistical methods

We analysed the trendwise development in individual age groups using models, assuming that the natural logarithm of the incidence was a function of time. We used both linear models and models including a quadratic (trend) term. While the linear models assume a constant growth rate in incidence, the second-order polynomial models allow a non-linear development of a certain kind. Although the second-order model may not give a completely accurate description of the trendwise development, it makes it possible to test whether allowing a specific type of non-linearity improves the linear model. A more detailed picture of the trends is given in graphs for 10-year age groups. A *P*-value (two sided) of 0.05 or lower was considered to be statistically significant. Age-standardized rates were calculated using the Swedish population in 1970.

**Figure 1** Incidence of breast cancer in Sweden 1984–93. Number of new cases of breast cancer per 100 000 women, by 10-year age groups and age standardized (to the Swedish population in 1970). -□-, 0–39 years; --▲--, 40–49 years; - -+- -, 50–59 years; - -●- -, 60–69 years; - -×- -, 70–79 years; ——, 80–99 years; - - -, age standardized

### RESULTS

Figure 1 demonstrates the trends in breast cancer incidence by age groups. The age-standardized breast cancer incidence increased from 91 per 100 000 women-years in 1984 to a maximum of 115 in 1990, thereafter diminishing to 104 per 100 000 in 1993. Women under the age of 40 years had no notable change in incidence over time. In contrast women aged 40–49 years had a seemingly steady increase from the late 1980s. Among 50 to 69-year-old women, we observed a substantial increase from the late 1980s followed by a decline in the early 1990s. For women 70 years and older, no clear patterns were noted.

Further analyses using linear models demonstrated an average annual increase in the age-standardized rate of 2.1% (95% confidence interval 0.9–3.3) (Table 1). The second-order term in the non-linear model was significant ($P = 0.04$) with a negative sign, confirming the non-linear development – an early increase followed by a decrease – in the age-standardized rate. The age-specific analyses showed no clear trends among women below 40 years of age, as reported in Table 1. For the aggregated group of women aged below 40 years, the annual age-standardized change was –1.4% (95% CI –2.7–0.0). In contrast, there was a significant increase for all 5-year age groups between 40 and 69 years.

We noted a particularly pronounced increase among women 50–69 years of age, with average annual increments of 3.3% to 4.3%. In these age groups, the most rapid increase started a few years into the 10-year study period, with indications of decreasing trends towards the end of the period (Figure 1). For women aged 55–69 years, the latter trends were supported in the modelling allowing for nonlinear effects, with negative but statistically non-significant second-order terms ($P = 0.07–0.15$). Among women 70 years or older, we found no clear patterns, except for the age group 70–74 years in which there was a marked non-linear trend, an initial increase followed by a later decline.

### DISCUSSION

Our analysis revealed three notable patterns: (1) compared with the 31-year period 1958 through 1988, the average annual rate of increase (age-standardized) was somewhat higher, 2.1% vs 1.3%; (2) women below age 40 years showed no clear change in incidence (if anything a tendency towards a decrease), whereas in the previous study a declining rate of increase was found; and (3) most importantly, women aged 40–69 years showed a pronounced but seemingly transient rise from the late 1980s.

A comparison of contemporaneous breast cancer incidence patterns in Norway and Sweden is potentially informative. In contrast to Sweden, the Norwegian data, unaffected by mammographic screening, indicated a 4% annual increase in the incidence for women below 50 years of age. We found little change in the

**Table 1** Trends in age-specific and age-standardized breast cancer incidence rates in Sweden 1984–93

| Age (years) | Linear model | | Model including a quadratic-trend term | |
|---|---|---|---|---|
| | Annual change in % | P-value | Sign of the second-order term | P-value of the second-order term |
| 25–29 | –4.6 | 0.25 | + | 0.24 |
| 30–34 | 0.0 | 0.99 | + | 0.89 |
| 35–39 | –1.2 | 0.39 | – | 0.35 |
| 40–44 | 1.8 | 0.04 | – | 0.74 |
| 45–49 | 2.0 | 0.005 | – | 0.22 |
| 50–54 | 4.3 | 0.001 | + | 0.94 |
| 55–59 | 3.5 | 0.007 | – | 0.07 |
| 60–64 | 3.3 | 0.007 | – | 0.15 |
| 65–69 | 4.0 | 0.014 | – | 0.10 |
| 70–74 | 0.4 | 0.64 | – | 0.004 |
| 75–79 | –1.6 | 0.012 | – | 0.57 |
| 80–84 | 1.0 | 0.06 | – | 0.54 |
| 85– | –1.4 | 0.08 | + | 0.25 |
| Age standardized | 2.1 | 0.004 | – | 0.04 |

 © Cancer Research Campaign 1998

incidence for women below 40 years, but a slight (up to 2%) rise in women aged 40–49 years of age. The absence of trends among the youngest women in Sweden contradict the trend in Norway being caused by oral contraceptives (Matheson and Tretli, 1996). As these have been used at least as extensively in Sweden (Lund et al, 1989), their risk-increasing effect, which appears to be transient (Collaborative Group, 1996), should therefore be the most marked at young ages. Moreover, trends in fertility patterns and exposure to other established risk factors for breast cancer are probably similar in the two countries – except for factors linked to birth cohorts that were adolescent during the Second World War (Tretli and Gaard, 1996).

In Sweden, the estimated proportion of hormone replacement therapy (HRT) users in perimenopausal age groups was 20% in the late 1980s (Lindgren et al, 1993) compared with less than 10% in the late 1970s (Persson et al, 1983). However, the possible link between HRT and breast cancer risk is rather weak, with relative risk estimates of 1.5–2, and the risk relationship seems to be limited to exposure exceeding 6–10 years (Brinton and Schairer, 1993). Therefore, HRT is not likely to be an important explanation for the observed population trends.

Rather, we believe that mammographic screening – proven to be sensitive in women at ages above 50 years (Nyström et al, 1993) – provides the most plausible explanation (Fletcher et al, 1993) of the recent trends in Sweden. Population-based programmes were introduced in most counties of Sweden from 1987, with 20 of 26 counties included in 1989 and participation rates among invited women averaging 81% in the period 1993–95 (Olsson et al, 1995). The pattern of a rise in incidence in the age groups 50–69 years in the late 1980s followed by a decline in the 1990s is consistent with early detection of prevalent cancers through mammography screening, as earlier demonstrated in the USA (Chu et al, 1996).

We conclude that the recent breast cancer incidence trends in Sweden are largely reassuring and attributable to the introduction of population-based screening with mammography. In contrast, increasing incidence among young women in Norway cannot be readily explained and deserves, therefore, further investigation.

### ACKNOWLEDGEMENT

This study was supported by grants from the Swedish Cancer Society.

### REFERENCES

Brinton A and Schairer C (1993) Estrogen replacement therapy and breast cancer risk. *Epidemiol Rev* **15**: 66–79

Cancer Registry (1996) *Cancer Incidence in Sweden 1993*. National Board of Health and Welfare: Stockholm

Chu KC, Tarone RE, Kessler LG, Lynn AG, Ries LAG, Hankey BF, Miller BA and Edwards BK (1996) Recent trends in US breast cancer incidence, survival, and mortality rates. *J Natl Cancer Inst* **88**: 1571–1579

Collaborative Group on Hormonal Factors in Breast Cancer (1996) Breast cancer and hormonal contraceptives: collaborative reanalyses of individual data on 53,297 women with breast cancer and 100,239 women without breast cancer from 54 epidemiological studies. *Lancet* **347**: 1713–1727

Ewertz M and Carstensen B (1988) Trends in breast cancer incidence and mortality in Denmark 1943–1982. *Int J Cancer* **41**: 46–51

Fletcher SW, Black W, Harris R, Rimer BK and Shapiro S (1993) Report of the International Workshop on Screening for Breast Cancer. *J Natl Cancer Inst* **85**: 1644–1656

Holford TR, Roush GC and McKay LA (1991) Trends in female breast cancer in Connecticut and the United States. *J Clin Epidemiol* **44**: 29-39

Lindgren R, Berg G, Hammar M and Zuccon E (1993) Hormonal replacement therapy and sexuality in a population of Swedish postmenopausal women. *Acta Obstet Gynecol Scand* **72**: 292–297

Lund E, Meirik O, Adami H-O, Bergström R, Christoffersen T and Bergsjø P (1989) Oral contraceptive use and premenopausal breast cancer in Sweden and Norway: possible effects of different pattern of use. *Int J Epidemiol* **18**: 527–532

Matheson I and Tretli S (1996) Changes in breast cancer incidence among Norwegian women under 50. *Lancet* **348**: 900–901

Mattsson B and Wallgren A (1984) Completeness of the Swedish Cancer Register – non-notified cases on death certificates in 1978. *Acta Radiol Oncol* **23**: 305

Nyström L, Rutquist L, Wall S, Lindgren A, Lindquist M, Rydén S, Andersson I, Bjurstam N, Fagerberg G, Frisell J (1993) Breast cancer screening with mammography: overview of Swedish randomized trials. *Lancet* **341**: 973–978

Olsson S, Andersson I, Bjurstam N, Frodis E, Håkansson S, Lithander E and Karlberg I (1995) 600 000 women are examined by mammography each year (in Swedish). *Lakartidningen* **92**: 552–556

Persson I, Adami H-O, Lindberg BS, Johansson EDB and Manell P (1983) Practice and patterns of oestrogen treatment in climacteric women in a Swedish population. A descriptive epidemiological study. Part I. *Acta Gyn Obstet Scand* **62**: 289–296

Persson I, Bergström R, Sparén P, Thörn M and Adami H-O (1993) Trends in breast cancer incidence in Sweden 1958–1988 by time period and birth cohort. *Br J Cancer* **68**: 1247–1253

Quinn M and Allen E (1995) Changes in incidence of and mortality from breast cancer in England and Wales since introduction of screening. *Br Med J* **311**: 1391–1395

Tretli S and Gaard M (1996) Lifestyle changes during adolescence and risk of breast cancer: an ecological study of the effect of World War II in Norway. *Cancer Causes Control* **7**: 517–521

© Cancer Research Campaign 1998